# The synthesis and characterization of a series of cocrystals of an isoniazid derivative with butan-2-one and propan-2-one

**DOI:** 10.1107/S2053229623007179

**Published:** 2023-08-21

**Authors:** Matthew Clarke Scheepers, Andreas Lemmerer

**Affiliations:** a Molecular Sciences Institute, School of Chemistry, University of the Witwatersrand, Private Bag 3, Johannesburg, Gauteng 2050, South Africa; Wilfrid Laurier University, Waterloo, Ontario, Canada

**Keywords:** crystal structure, cocrystal, isoniazid, di­carb­oxy­lic acid, pyridine, carbohydrazide, naphthoic acid, thermal analysis

## Abstract

Four cocrystals containing *N*′-(butan-2-yl­idene)pyridine-4-carbohydrazide (**izbt**) and one cocrystal containing *N*′-iso­propyl­ideneisonicotinohydrazide (**izact**) were synthesized by reacting isoniazid with either butan-2-one (for the former) or acetone (for the latter). The coformers used to synthesize the **izbt** cocrystals were 2,4-di­hydroxy­benzoic acid, 2,5-di­hydroxy­benzoic acid, 2-chloro-4-nitro­benzoic acid and 1-naphthoic acid. 1-Naphthoic acid was also used with **izact** to form a cocrystal. The 1:1 cocrystals are: *N*′-(butan-2-yl­idene)pyridine-4-car­bohydrazide–1-naphthoic acid (**izbt**–**1nta**), C_10_H_13_N_3_O·C_11_H_8_O_2_, *N*′-(butan-2-yl­idene)pyridine-4-carbohydrazide–2,4-di­hydroxy­benzoic acid (**izbt**–**2,4-dhba**), C_10_H_13_N_3_O·C_7_H_6_O_4_, *N*′-(propan-2-yl­idene)pyridine-4-carbohydrazide–1-naphthoic acid (**izact**–**1nta**), C_9_H_11_N_3_O·C_11_H_8_O_2_, *N*′-(butan-2-yl­idene)pyridine-4-carbohydrazide–2-chloro-4-nitro­benzoic acid (**izbt**–**2c4n**), C_10_H_13_N_3_O·C_7_H_4_ClNO_4_, and *N*′-(butan-2-yl­idene)pyridine-4-carbohydrazide–2,5-di­hy­droxy­benzoic acid (**izbt**–**2,5-dhba**), C_10_H_13_N_3_O·C_7_H_6_O_4_. The cocrystals con­taining **izbt** were com­pared to those containing the same (or similar) coformers with **izact** that have been reported either here or in the Cambridge Structural Database (CSD). Most of the cocrystals showed different packing despite having the same hydrogen-bonding motifs. The cocrystals were characterized by single-crystal X-ray diffraction (SC-XRD), powder X-ray diffraction (PXRD) and differential scanning calorimetry (DSC).

## Abstract 2

Four cocrystals containing *N*′-(butan-2-yl­idene)pyridine-4-carbohydrazide and one cocrystal containing *N*′-iso­propyl­ideneisonicotinohydrazide were synthesized by reacting isoniazid with either butan-2-one (for the former) or acetone (for the latter). The coformers used to synthesize these cocrystals were 2,4-di­hydroxy­benzoic acid, 2,5-di­hydroxy­benzoic acid, 2-chloro-4-nitro­benzoic acid and 1-naphthoic acid. The cocrystals were characterized by SC-XRD, PXRD and DSC.

## Introduction

In the pharmaceutical industry, it is often typical for new and existing drugs to have poor physicochemical properties. The poor performance from these drugs can hamper their success on the market. The modification of an existing drug can yield a new product with possibly improved properties compared to the original drug mol­ecule. Although this would require new clinical trials, it could prove to be advantageous for long-term success. From a crystal engineering perspective, this could prove to be an opportunity to explore new solid-state structural landscapes.

One approach of crystal engineering with repsect to changing the solid-state form of active pharmaceutical ingredients (APIs) is to use cocrystals. Although no universal definition of a cocrystal exists, several different definitions have been proposed by different authors. The definition proposed by Aitipamula *et al.* (2012 ▸) is as follows: ‘cocrystals are solids that are crystalline single phase materials com­posed of two or more different mol­ecular and/or ionic com­pounds generally in a stoichiometric ratio.’ Grothe defines a cocrystal as ‘a crystal with a coformer mol­ecule plus either another coformer or at least two ions,’ with further classifications depending on whether the crystal also contains ions, solvate mol­ecules or water (Grothe *et al.*, 2016 ▸). Cocrystals have been proven to have new properties, such as having a different solubility or bioavailability over the starting material (Karimi-Jafari *et al.*, 2018 ▸). It can even lead to the possibility of having multiple drugs in one crystal form (Wang *et al.*, 2021 ▸). Unfortunately, cocrystal design and synthesis is not straightforward; it is possible to fail creating a cocrystal despite using a reasonable cocrystal design methodology (Bučar *et al.*, 2013 ▸). 
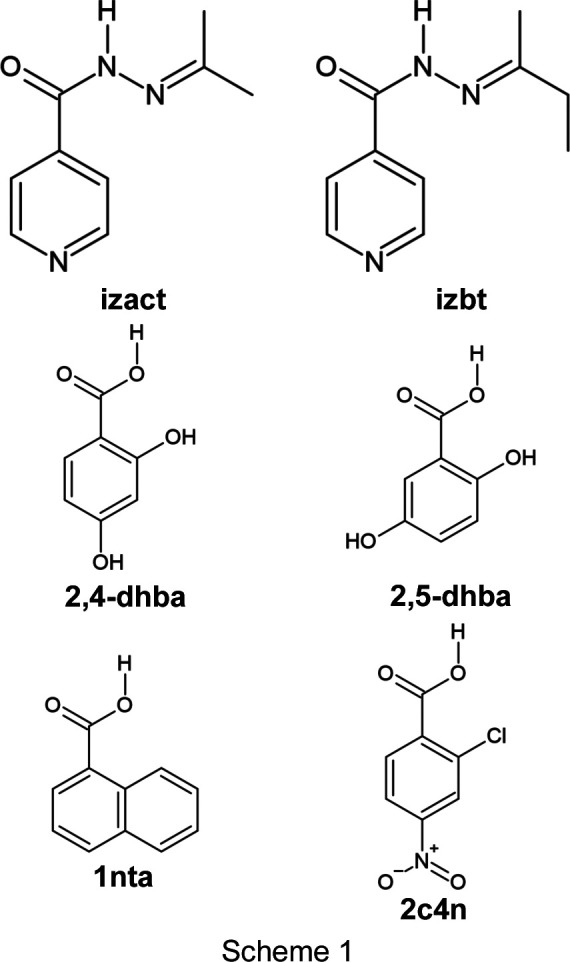


Isoniazid (**inh**) is an anti­bacterial drug used to treat *Mycobacterium tuberculosis* bacteria (TB). It is often combined with several different drugs in a fixed-dose combination (FDC) as part of the treatment for this disease (Murray *et al.*, 2015 ▸). However, **inh** has been known to undergo degradation in the presence of other drugs (Bhutani *et al.*, 2005 ▸). **Inh** is a fairly simple drug mol­ecule, consisting of a pyridine ring, an amide group and a hydrazine group. One way to modify **inh** is to employ a Schiff base reaction using **inh** and either an aldehyde or a ketone. In previous work, we modified isoniazid with acetone, butan-2-one, 4-hy­droxy-4-methyl­pentan-2-one and benzo­phenone, and explored a different number of cocrystal and mol­ecular salt crystal structures (Lemmerer *et al.*, 2010 ▸; Madeley *et al.*, 2019 ▸; Scheepers & Lem­merer, 2022 ▸; Lemmerer, 2012 ▸). In particular, a decent number of cocrystals and mol­ecular salts with isoniazid derived from acetone and butan-2-one have been reported, with seven crystal structures containing *N*′-(butan-2-yl­idene)pyridine-4-carbohydrazide (butan-2-one-based derivative, **izbt**) and 15 containing *N*′-iso­propyl­idene­iso­nico­tino­hy­dra­zide (acetone-based derivative, **izact**). Out of these, only five pairs share the same coformer, of which, three pairs are isostructural (Table 1 ▸). It should not be too surprising to find crystal structures becoming isostructural when certain functional groups are exchanged with a similar one, for example, changing a chlorine to a bromine or methyl to an amino group (Clarke *et al.*, 2012 ▸). However, it is still possible that exchanging one functional group for another can yield a com­pletely different structure. In the case of **izact** and **izbt**, the difference is the presence or absence of the methyl­ene group in the alkyl group. In three of the cases, this had no effect, but in the case of the anyhydrous forms of **izact**– and **izbt**–3-hy­droxy­benzoic acid, and **izact**– and **izbt**–2-hydro­benzoic acid, there was a significant difference in the packing. Based on the structures listed in Table 1 ▸, we would expect that the methyl­ene group would very likely have a small or even insignificant effect on the overall packing of the mol­ecules in the crystal structure; however, with a small sample size it is difficult to assess if this is a reasonable assertion. Therefore, the aim of this work is to expand the number of multi­com­ponent crystal structure pairs and com­pare them, in order to confirm whether the addition of the methyl­ene group can have a significant impact of the packing of these structures. The simplest way to achieve this is to expand the number of **izbt** cocrystals using coformers that worked for **izact**; the coformers used include: 2,4-di­hydroxy­benzoic acid (**2,4-dhba**), 2,5-di­hydroxy­benzoic acid (**2,5-dhba**) and 2-chloro-4-nitro­benzoic acid (**2c4n**). In addition, cocrystals containing 1-naphthoic acid (**1nta**) with **izact** and **izbt**, respectively, were synthesized and characterized. **1nta** was used to observe the effect of using a bulky double ring as opposed to using coformers consisting of a single ring. The structures of these com­pounds are represented in Scheme 1.

## Experimental

### Materials

All materials were purchased from Sigma–Aldrich and were used without further purification.

### General procedure for the synthesis of izbt and izact cocrystals

The general procedure for synthesizing cocrystals featuring either **izbt** or **izact** is as follows: stoichiometric ratios of **inh** and the respective coformer (1:1 ratio) were dissolved in absolute ethanol (5 ml) in a small vial. The deriving ketone (acetone or butan-2-one) (1 ml) was added. This vial was closed and the mixture stirred for 4 h. Afterwards, the lid on the vial was replaced with a lid with a hole in it and the vial kept in a dark cupboard. After several days, crystals remained after the solvent evaporated.

### Powder X-ray diffraction (PXRD)

PXRD was used to determine the bulk phase purity of each sample. PXRD data for all forms were measured at 293 K on a Bruker D2 Phaser diffractometer which employs a sealed tube Co X-ray source (λ = 1.78896 Å), operating at 30 kV and 10 mA, and a LynxEye PSD detector in Bragg–Brentano geometry. The powder patterns for the cocrystals are pre­sented in the supporting information, where the experimentally measured pattern is com­pared to the calculated patterns obtained from the single-crystal X-ray diffraction (SC-XRD) data, as well as the calculated patterns of its com­ponents using data from the Cambridge Structural Database (CSD, Version 2022.1.0; Groom *et al.*, 2016 ▸).

### Single-crystal X-ray diffraction (SC-XRD) and refinement

Crystal data, data collection and structure refinement details are summarized in Table 2 ▸. All carbon-bound H atoms were placed in idealized positions and refined as riding atoms, with *U*_iso_(H) parameters 1.2 or 1.5 times those of the parent atoms. Most nitro­gen- and oxygen-bound H atoms were located in difference Fourier maps, and their coordinates and isotropic displacement parameters were refined freely.

### The Cambridge Structural Database (CSD)

The CSD (Version 2022.1.0; Groom *et al.*, 2016 ▸) was used to com­pare the cocrystals presented in this work with the cocrystals of **izact**. The only restriction was that entries must be classified as being organic. *Mercury* (Macrae *et al.*, 2020 ▸) was used to inspect the crystal structures. The Crystal Structure Similarity tool was used to com­pare the structural similarity of selected structures.

### Differential scanning calorimetry (DSC)

DSC data were collected using a Mettler Toledo DSC 3 with aluminium pans under nitro­gen gas (flow rate = 10 ml min^−1^). Exothermic events were shown as peaks. Samples were heated and cooled to determine the melting points, as well as any additional phase transitions. The tem­per­ature and energy cali­brations were performed using pure indium (purity 99.99%, m.p. 156.6 °C, heat of fusion: 28.45 J g^−1^) and pure zinc (purity 99.99%, m.p. 479.5 °C, heat of fusion: 107.5 J g^−1^). Samples were heated to 250 °C from 25 °C before being cooled back down to 25 °C at a heating or cooling rate of 10 °C min^−1^.

## Results and discussion

### Synthesis of cocrystals

**Figure 1:**
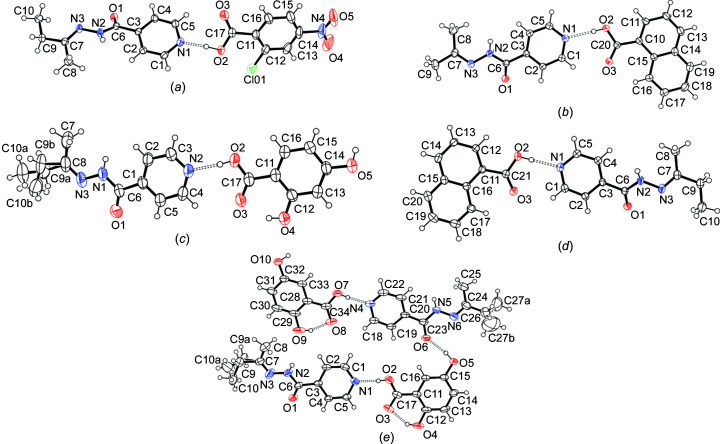
The mol­ecular structures of (*a*) **izbt**–**2c4n**, (*b*) **izact**–**1nta**, (*c*) **izbt**–**2,4-dhba**, (*d*) **izbt**–**1nta** and (*e*) **izbt**–**2,5-dhba**. Displacement ellipsoids are drawn at the 50% probability level and H atoms are shown as small spheres of arbitrary radii.

In this work, six cocrystals were synthesized and characterized. Five of these cocrystals contained **izbt** and one contained **izact**. The four coformers chosen were: 2,4-di­hydroxy­benzoic acid (**2,4-dhba**), 2,5-di­hydroxy­benzoic acid (gentisic acid, abbreviated as **2,5-dhba**), 2-chloro-4-nitro­benzoic acid (**2c4n**) and 1-naphthoic acid (**1nta**). The first three coformers were chosen because previous cocrystals containing said coformers with **izact** had been synthesized and characterized previously, and it would be a good reference to com­pare the respective corystals. **1nta** was chosen due to its naphthalene ring, as it would be inter­esting to see if its bulky nature had an effect on the overall packing. These cocrystals are described below, together with that of **izbt** com­pared to its **izact** counterpart, as well as any other notable cocrystal of **izact**. The crystal structure data are given in Table 2 ▸, while the displacement ellipsoid plots are shown in Fig. 1 ▸. Hydrogen-bond tables can be found in the supporting information.

Powder patterns were collected for each sample and were com­pared to the powder patterns calculated from the single-crystal structural data. These powder patterns are also com­pared to the powder patterns of each of the com­ponents, including the polymorphic forms of the com­ponents which are polymorphic. It should be noted that we are com­paring the hydrated form of **izbt** to the powder patterns of the cocrystals presented here. This was done because it was the closest form to a ‘pure’ com­ponent we can get, and in our own investigations, we have obtained the hydrated form of **izbt** exclusively when using the same crystallizing conditions we used to obtain the cocrystals presented here, which indicates that **izbt** is most likely highly hygroscopic, obtaining the water from either the waters of reaction or atmospheric moisture, or both. This com­parison between the experimental and calculated patterns was made to confirm the bulk phase purity. These patterns are presented in the supporting information.

### Crystal structure of izbt–2,4-dhba

**Figure 2:**
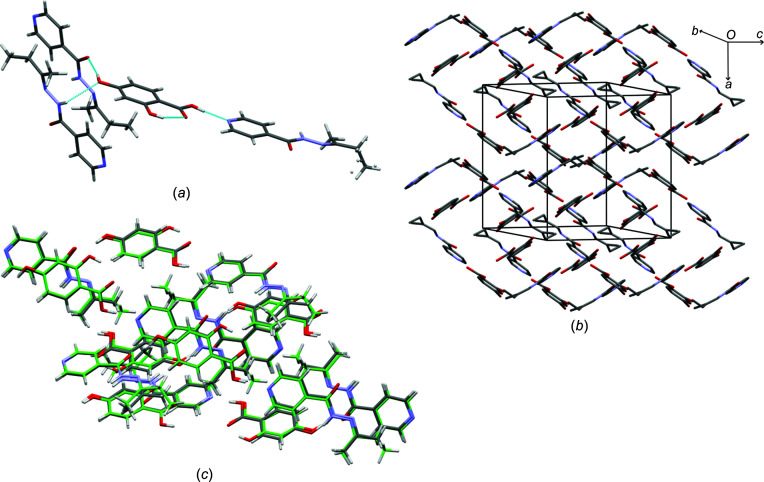
The crystal structure of **izbt**–**2,4-dhba**, showing (*a*) the hydrogen bonding present in the structure, (*b*) the packing of the structure (with H atoms omitted) and (*c*) an overlay of **izbt**–**2,4-dhba** with **izact**–**4hba**, showing their isostructurality.

**Izbt** formed a cocrystal with **2,4-dhba** which crystallized as colourless blocks in the space group *P*2_1_/*n*, with the asymmetric unit consisting of one mol­ecule each of **izbt** and **2,4-dhba**. A disorder model is present, where two C atoms (C9 and C10) of the alkyl portion of **izbt** were split over two different positions, respectively. The pyridine ring of **izbt** forms a hydrogen bond with the carb­oxy­lic acid group of **2,4-dhba**, while the hy­droxy group at the 4-position of the **2,4-dhba** mol­ecule (O5—H5) forms two different hydrogen bonds with the amide group of **izbt**, one where the hy­droxy group is the hydrogen-bond donor, forming a hydrogen bond with the O atom of the amide group [O5—H5⋯O1^i^; symmetry code: (i) *x* + 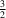 , −*y* + 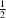 , *z* + 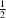 ], and one where the hy­droxy group is the hydrogen-bond acceptor, forming a hydrogen bond with the amine portion of the amide group [N1—H1⋯O5^ii^; symmetry code: (ii) −*x* + 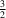 , *y* + 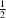 , −*z* + 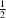 ] [Fig. 2 ▸(*a*)]. This hydrogen-bond arrangement ultimately forms a tetra­mer with an 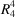 (12) ring hydrogen-bond motif. The hy­droxy group at the 2-position of **2,4-dhba** does not form any strong hydrogen bonds other than the typical intra­molecular hydrogen bond with the carb­oxy­lic acid group. The overall packing of the structure resembles a herringbone-type structure [Fig. 2 ▸(*b*)].

Although there is no equivalent cocrystal featuring **2,4-dhba** and **izact**, the crystal structure of **izbt**–**2,4-dhba** may be com­pared to that of **izact**–**4hba** (CSD refcode LATKOI). Fig. 2 ▸(*c*) shows the overlay of these two structures using the Crystal Structure Similarity tool of *Mercury* (Macrae *et al.*, 2020 ▸). The crystal structure parameters of **izact**–**4hba** match closely with those of **izbt**–**2,4-dhba**, and when the Crystal Structure Similarity tool of *Mercury* was used, it showed that the respective mol­ecules matched up well, indicating that they were isostructural. This implies that, in this case, the hy­droxy group at the 2-position and the extra methyl group on the alkyl chain did not have any significant effect on the overall packing on the structure.

### Crystal structure of izbt–2,5-dhba

**Figure 3:**
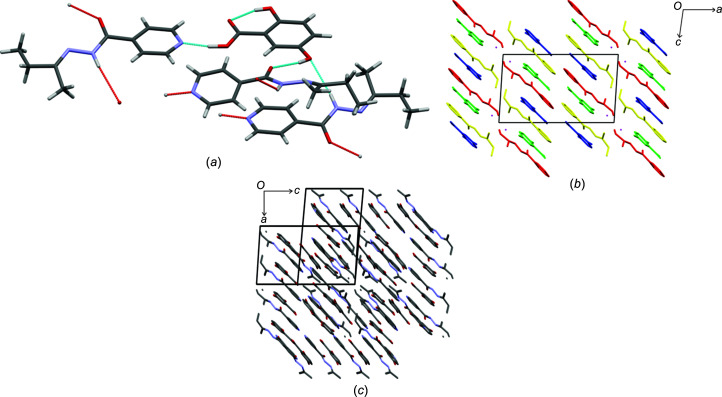
The crystal structure of **izbt**–**2,5-dhba**, showing (*a*) the hydrogen bonding present, (*b*) the packing (with H atoms omitted, **izbt** in blue and green, and **2,5-dhba** in red and yellow) and (*c*) the overlay of **izact**–**2,5-dhba** and **izbt**–**2,5-dhba**.

**Izbt** formed a cocrystal with **2,5-dhba** which crystallized as colourless blocks in the space group *P* 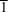 , with the asymmetric unit consisting of two molecules each of **izbt** and **2,5-dhba**. A disorder model exists where the methylene group of one of the **izbt** molecules has been split into two different sites (C26*A* and C26*B*). Like **izbt**–**2,4-dhba**, the corystals of **izbt**–**2,5-dhba** exhibit a similar hydrogen-bonding trend: a hydrogen bond is formed between the pyridine ring of **izbt** and the carboxylic acid group of **2,5-dhba**, while the hydroxy group at the 5-position of **2,5-dhba** forms a 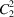 (11) chain–ring hydrogen-bond motif with the O and H atom of the amide group of adjacent **izbt** molecules [Fig. 3 ▸(*a*)]. The overall packing is a layered-type structure, with the alkyl group separating the ring layers [Fig. 3 ▸(*b*)]. In comparison, the structure of **izact**–**2,5-dhba** (refcode NAKYOQ; Oruganti *et al.*, 2016 ▸) is very similar to its **izbt** counterpart. Both exhibit the same hydrogen-bond pattern, as well as the overall packing pattern, and the reduced unit-cell lengths of NAKYOQ are comparable (*a* = 9.119, *b* = 11.581 and *c* = 15.273 Å) [Fig. 3 ▸(*c*)].

### Crystal structure of izbt–2c4n

**Figure 4:**
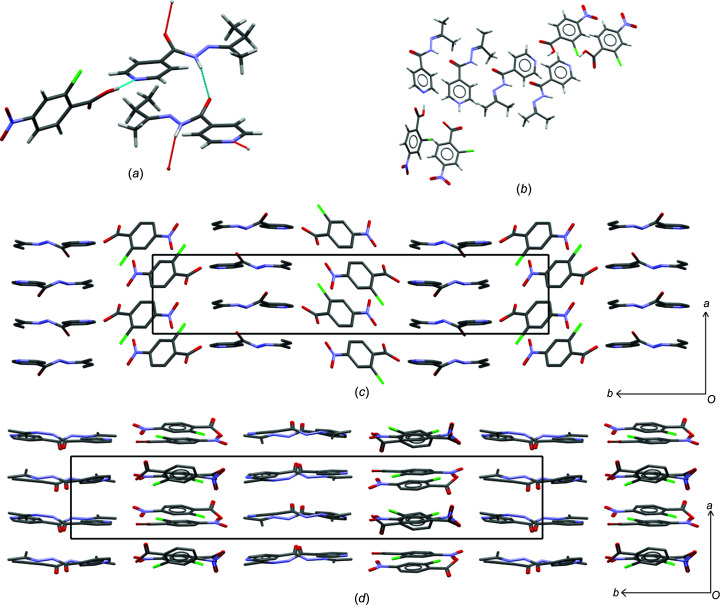
The crystal structure of **izbt**–**2c4n** and **izact**–**2c4n** (CSD refcode LATLID), showing (*a*) the hydrogen bonding present in **izbt**–**2c4n**, (*b*) the asymmetric unit of **izact**–**2c4n**, (*c*) the packing of **izbt**–**2c4n** (with H atoms omitted for clarity) and (*d*) the packing of **izact**–**2c4n** (with H atoms omitted for clarity).

**Izbt** and **2c4n** formed a cocrystal, crystallizing as yellow plates. The asymmetric unit consists of one mol­ecule each of **izbt** and **2c4n**, crystallizing in the space group *P*2_1_/*n*. The hydrogen bonding observed in **izact**–**2c4n** consists of the typical scheme observed for these types of structures: the carb­oxy­lic acid group of **2c4n** forms a hydrogen bond with the pyridine ring of **izbt**, while the amide group of **izbt** forms a *C*(4) chain hydrogen-bond motif between the H atom of the amide group and the O atom of the amide group of another **izbt** mol­ecule [Fig. 4 ▸(*a*)]. This chain hydrogen-bond motif causes the mol­ecules of **izbt** to lie almost perpendicular with respect to each other in an alternating pattern, and extends along the *a* and *c* axes. This ultimately forms a series of ribbons which pack together to form the crystal structure observed in Fig. 4 ▸(*c*).

The **izact**–**2c4n** cocrystal (refcode LATLID) is much different in com­parison. According to Grothe *et al.* (2016 ▸), this crystal system can be defined as a ‘cocrystal salt’, since its asymmetric unit consists of three neutral mol­ecules of **2c4n** and one ion of **2c4n**, with three neutral mol­ecules of **izact** and one protonated ion of **izact** [Fig. 4 ▸(*b*)]. This crystal structure crystallizes in the chiral space group *P*2_1_. Like the structure of **izbt**–**2c4n**, mol­ecules and ions of **izact** are connected to each other *via* a series of 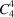 (16) chain hydrogen-bond motifs involving the H atom of the amide group forming a hydrogen bond with the O atom of the amide group from another **izact** mol­ecule. The carb­oxy­lic acid group of **2c4n** also forms a hydrogen bond with the pyridine ring of **izact** (charge-assisted for the cation–anion pair). The overall packing of **izact**–**2c4n** also consists of ribbons, but these differ from the structure of **izbt**–**2c4n** [Fig. 4 ▸(*d*)]. Unlike in the crystal structure of **izbt**–**2c4n**, the arrangement of the **izbt**–**2c4n** bonded pairs lie almost parallel to each other, instead of the rotation of almost 90° seen in **izbt**–**2c4n**.

### Crystal structures of izact–1nta and izbt–1nta

**Figure 5:**
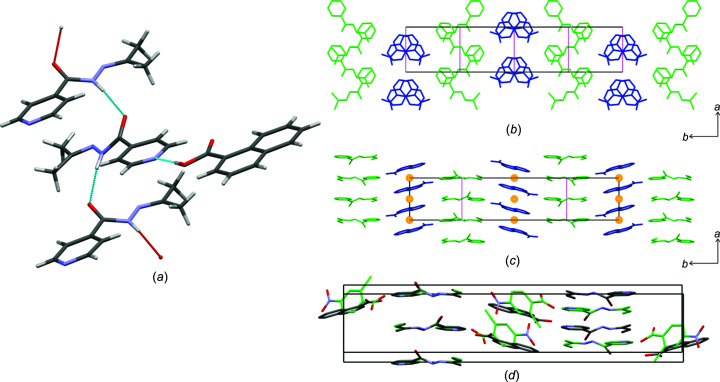
(*a*) The hydrogen bonding present in **izact**–**1nta**, showing the typical hydrogen bonding in the structures of **izact**–**1nta** and **izbt**–**1nta**. The packing present in the crystal structure of (*b*) **izact**–**1nta** and (*c*) **izbt**–**1nta**, with the H atoms omitted for clarity. In parts (*b*) and (*c*), the respective isoniazid derivative is presented in green, **1nta** in blue, glide planes as magenta lines and inversion centres as orange spheres. (*d*) The overlap of **izbt**–**1nta** and **izbt**–**2c4n** (mol­ecules in green), showing that the structures are not isostructural.

**Izact** and **izbt** each formed a cocrystal with **1nta**, both crystallizing as colourless plates. Despite sharing similar unit-cell parameters, the structures are not isostructural, as the crystal structure of **izact**–**1nta** crystallizes in the space group *Cc*, while the crystal structure of **izbt**–**1nta** crystallizes in the space group *P*2_1_/*n*. These structures are also not isostructural with **izbt**–**2c4n**, despite sharing similar unit-cell parameters, as can be seen in Fig. 5 ▸(*d*). Both crystal structures share the same hydrogen-bonding patterns. **1nta** forms a hydrogen bond with the isoniazid derivative, while the isoniazid derivative forms a *C*(4) chain hydrogen-bond motif involving the H atom of the amide group and the O atom of the amide group from a neighbouring mol­ecule of the isoniazid derivative [Fig. 5 ▸(*a*)], which expands generally in the direction of the *a* axis as ribbons. The key difference between the two structures lies in the packing, where the difference between the *P*2_1_/*n* and *Cc* space groups is that the inversion centres in *P*2_1_/*n* [Fig. 5 ▸(*b*)] are replaced by glide planes in *Cc* [Fig. 5 ▸(*c*)]. This changes the orientation of the mol­ecules existing in both structures with respect to the unit cell. Overall, the packing of both structures may be described as herringbone.

### Thermal analysis

**Figure 6:**
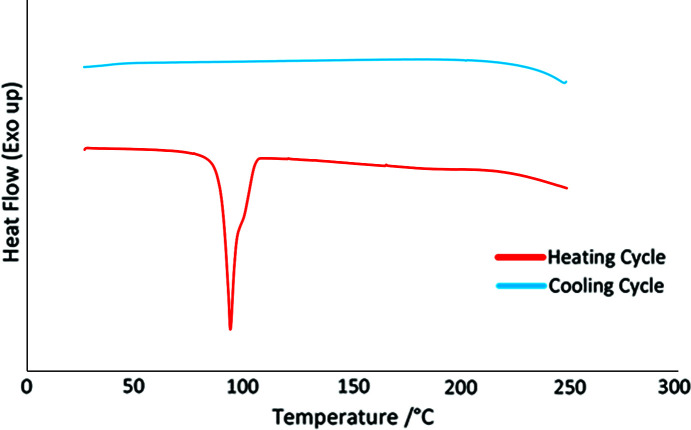
DSC curve for **izbt**–**1nta**, representing the typical DSC curve observed for each of the cocrystals.

DSC curves were collected for all ten cocrystals. The DSC curve of **izbt**–**1nta** is presented in Fig. 6 ▸ as a representative curve, while the remaining DSC curves can be found in the supporting information. The onset and enthalpy values are presented in Table 3 ▸. The melting points and enthalpies of some of the cocrystals related to the cocrystals presented in this work are also included. In each of the curves, only one large distinct peak was observed on the heating stage, which correlates to the melting/decom­position of the sample. No peaks were observed on the cooling stage of the DSC curves, indicating that no recrystallization occurred. A common feature between the cocrystals is that their melting points are much lower than the melting points of the acid coformers. This makes sense since the 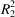 (8) hydrogen-bond ring motif formed between the carb­oxy­lic acid pairs is expected to be stronger than that formed by carb­oxy­lic acid–pyridine. From a pharmaceutical point of view, a lower melting point is usually indicative of a product that has a better drug solubility, which indicates the possibility of having better pharmaceutical properties compared to their individual com­ponents (Chu & Yalkowsky, 2009 ▸). A comparison of the melting/decom­position points and enthalpies of the **izact** cocrystals with those of their **izbt** counterparts indicates that the values tend to be similar to each other, which would make sense considering their overall similar hydrogen-bond schemes.

## Conclusions

Four cocrystals containing **izbt** and one cocrystal containing **izact** were synthesized and characterized. The structures of the cocrystals containing **izbt** were com­pared to their **izact** counterparts, except for **izbt**–**2,4-dhba**, which was instead com­pared to **izact**–**4hba**. Most of the structures of the cocrystals of **izbt** were different com­pared to their **izact** counterparts in terms of packing, despite sharing similar hydrogen-bond patterns. This would imply that the presence or absence of a methylene group can have a significant impact on the overall crystal structure packing, contrary to our initial assumption that the methyl­ene group has a small or even insignificant effect on the packing of mol­ecules in the crystal structure. The melting/decom­position points were found to be much lower than those of the coformers. The overall result shows that many crystal systems are temperamental: small differences between mol­ecules can lead to big changes in the overall packing.

## Supplementary Material

Crystal structure: contains datablock(s) izbt1nta, izbt24dhba, izact1nta, izbt2c4n, izbt25dhba, global. DOI: 10.1107/S2053229623007179/dv3024sup1.cif

Structure factors: contains datablock(s) izbt1nta. DOI: 10.1107/S2053229623007179/dv3024izbt1ntasup2.hkl

Structure factors: contains datablock(s) izbt24dhba. DOI: 10.1107/S2053229623007179/dv3024izbt24dhbasup3.hkl

Structure factors: contains datablock(s) izact1nta. DOI: 10.1107/S2053229623007179/dv3024izact1ntasup4.hkl

Structure factors: contains datablock(s) izbt2c4n. DOI: 10.1107/S2053229623007179/dv3024izbt2c4nsup5.hkl

Structure factors: contains datablock(s) izbt25dhba. DOI: 10.1107/S2053229623007179/dv3024izbt25dhbasup6.hkl

Supporting information file. DOI: 10.1107/S2053229623007179/dv3024izbt1ntasup7.cml

Supporting information file. DOI: 10.1107/S2053229623007179/dv3024izbt24dhbasup8.cml

Supporting information file. DOI: 10.1107/S2053229623007179/dv3024izact1ntasup9.cml

Supporting information file. DOI: 10.1107/S2053229623007179/dv3024izbt2c4nsup10.cml

Supporting information file. DOI: 10.1107/S2053229623007179/dv3024izbt25dhbasup11.cml

Additional tables and figures. DOI: 10.1107/S2053229623007179/dv3024sup12.pdf

CCDC references: 2264437, 2264438, 2264434, 2264436, 2264435

## Figures and Tables

**Table 1:** Cocrystals of **izact** and **izbt** that share the same coformer The Crystal Structure Similarity tool of *Mercury* (Macrae *et al.*, 2020 ▸) was used to help determine if related structures were isostructural.

Coformer	**izact** refcode	**izbt** refcode	Isostructural
4-*tert*-Butyl­benzoic acid	GIYMAF	GIYMEJ	Yes
2-Hy­droxy­benzoic acid (salicylic acid)*	LATLAV	UBILAW	No
3-Hy­droxy­benzoic acid (anhydrous)	FADHUP	FADHOJ	No
3-Hy­droxy­benzoic acid hydrate	SAYPIT	SAYPOZ	Yes
4-Nitro­benzoic acid	XOPYEJ	XOPYUZ	Yes

Note: (*) it should be noted that the unit-cell parameters of both structures are similar but the orientations of the mol­ecules with respect to the unit cells differ significantly.

**Table:** For all structures: *Z* = 4. Experiments were carried out with Mo *K*α radiation using a Bruker D8 VENTURE PHOTON CMOS 100 area-detector diffractometer.

	**izbt**–**1nta**	**izbt**–**2,4-dhba**	**izact**–**1nta**
Crystal data			
Chemical formula	C_10_H_13_N_3_O·C_11_H_8_O_2_	C_10_H_13_N_3_O·C_7_H_6_O_4_	C_9_H_11_N_3_O·C_11_H_8_O_2_
*M*_r_	363.41	345.35	349.38
Crystal system, space group	Monoclinic, *P*2_1_/*n*	Monoclinic, *P*2_1_/*n*	Monoclinic, *C* *c*
Temperature (K)	173	173	173
*a*, *b*, *c* (Å)	7.4355 (13), 34.195 (6), 7.7242 (14)	11.0834 (6), 13.8364 (8), 12.0014 (7)	7.6312 (3), 33.5293 (12), 7.3493 (3)
α, β, γ (°)	90, 112.512 (4), 90	90, 115.710 (3), 90	90, 114.298 (1), 90
*V* (Å^3^)	1814.3 (6)	1658.26 (17)	1713.88 (12)
μ (mm^−1^)	0.09	0.10	0.09
Crystal size (mm)	0.34 × 0.28 × 0.12	0.32 × 0.25 × 0.21	0.72 × 0.33 × 0.08

Data collection			
Absorption correction	–	–	Multi-scan (*SADABS*; Sheldrick, 2001 ▸; Krause *et al.*, 2015 ▸)
*T*_min_, *T*_max_	–	–	0.684, 0.747
No. of measured, independent and observed [*I* > 2σ(*I*)] reflections	35501, 4511, 3552	25708, 4008, 1946	39441, 6819, 6145
*R*_int_	0.033	0.092	0.056
(sin θ/λ)_max_ (Å^−1^)	0.668	0.660	0.782

Refinement			
*R*[*F* ^2^ > 2σ(*F* ^2^)], *wR*(*F* ^2^), *S*	0.054, 0.142, 1.03	0.061, 0.219, 1.02	0.043, 0.122, 1.08
No. of reflections	4511	4008	6819
No. of parameters	254	248	238
No. of restraints	0	39	2
H-atom treatment	H atoms treated by a mixture of independent and constrained refinement	H-atom parameters constrained	H-atom parameters constrained
Δρ_max_, Δρ_min_ (e Å^−3^)	0.54, −0.34	0.70, −0.27	0.33, −0.17
Absolute structure	–	–	Flack *x* determined using 2626 quotients [(*I* ^+^) − (*I* ^−^)]/[(*I* ^+^) + (*I* ^−^)] (Parsons *et al.*, 2013 ▸)
Absolute structure parameter	–	–	0.0 (3)

**Table:** 

	**izbt**–**2c4n**	**izbt**–**2,5-dhba**
Crystal data		
Chemical formula	C_7_H_4_ClNO_4_·C_10_H_13_N_3_O	C_10_H_13_N_3_O·C_7_H_6_O_4_
*M*_r_	392.79	345.35
Crystal system, space group	Monoclinic, *P*2_1_/*n*	Triclinic, *P* 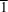
Temperature (K)	173	123
*a*, *b*, *c* (Å)	7.2682 (3), 34.0775 (15), 7.6124 (3)	9.2054 (3), 11.5589 (4), 15.6268 (6)
α, β, γ (°)	90, 111.081 (2), 90	92.383 (2), 93.092 (2), 90.666 (2)
*V* (Å^3^)	1759.27 (13)	1658.74 (10)
μ (mm^−1^)	0.26	0.10
Crystal size (mm)	0.46 × 0.26 × 0.11	0.45 × 0.38 × 0.13

Data collection		
No. of measured, independent and observed [*I* > 2σ(*I*)] reflections	111394, 5624, 5056	73254, 8026, 5972
*R*_int_	0.081	0.079
(sin θ/λ)_max_ (Å^−1^)	0.726	0.661

Refinement		
*R*[*F* ^2^ > 2σ(*F* ^2^)], *wR*(*F* ^2^), *S*	0.047, 0.129, 1.07	0.068, 0.191, 1.04
No. of reflections	5624	8026
No. of parameters	250	506
No. of restraints	0	21
H-atom treatment	H atoms treated by a mixture of independent and constrained refinement	H atoms treated by a mixture of independent and constrained refinement
Δρ_max_, Δρ_min_ (e Å^−3^)	0.41, −0.28	1.33, −0.68

Computer programs: *APEX2* (Bruker, 2012 ▸), *SAINT-Plus* (Bruker, 2012 ▸), *SHELXT2018* (Sheldrick, 2015*a* ▸), *SHELXL2018* (Sheldrick, 2015*b* ▸), *Mercury* (Macrae *et al.*, 2020 ▸), *ORTEP-3 for Windows* (Farrugia, 2012 ▸), *XPREP* (Bruker, 2012 ▸) and *WinGX* (Farrugia, 2012 ▸).

**Table 3:** Onset temperatures and associated enthalpies for the DSC curves of the cocrystals presented in this work, as well as the melting points and enthalpies of some of the com­parison cocrystals

Thermal event	Onset (°C)	Enthalpy (J g^−1^)	Enthalpy (kJ mol^−1^)
**Izact**–**1nta** melting/decom­position	106.1 ± 0.5	170.9 ± 5.2	59.7 **±** 2
**Izbt**–**1nta** melting/decom­position	89.5± 0.1	167.8 ± 2.5	61.0 ± 1
**Izbt**–**2c4n** melting/decom­position	102.1 ± 0.2	100.6 ± 2.3	39.5 **±** 1
**Izbt**–**2,4-dhba** melting/decom­position	174.8± 0.2	226.3 ± 4.3	78.2 ± 1
**Izbt**–**2,5-dhba** melting/decom­position	151.2 ± 0.2	132.5 ± 3.2	45.8 **±** 1
**Izact** m.p. (Lemmerer, 2012 ▸)	160.0	–	29.7
**Izact**–**2c4n** m.p. (Lemmerer, 2012 ▸)	93.4	–	33.4
