# Supplementary material for: The synthesis and characterization of a series of cocrystals of an isoniazid derivative with butan-2-one and propan-2-one
Source: Acta Crystallogr C Struct Chem. 2023 Aug 21;79(Pt 9):365–73. doi: 10.1107/S2053229623007179 (PMC10561595; doi:10.1107/S2053229623007179)
Supplement: Supplementary file 12 [file c-79-00365-sup12.pdf]

Acta Cryst C STRUCTURAL CHEMISTRY

Volume 79 (2023)

Supporting information for article:

The synthesis and characterization of a series of cocrystals of an isoniazid derivative with butan-2-one and propan-2-one

Matthew Clarke Scheepers and Andreas Lemmerer

S1 Hydrogen Bond tables

Table 1 Hydrogen-bond geometry (Å, º) for (izbt+1nta)

D—H···*A*	*D*—H	H···*A*	*D*···*A*	*D*—H···*A*
C1—H1···O3	0.95	2.51	3.208 (2)	130
C8—H8*C*···O1^{i}	0.98	2.62	3.128 (2)	112
N2—H2···O1^{i}	0.89 (2)	1.98 (2)	2.8732 (17)	174 (2)
O2—H2*B*···N1	0.87 (3)	1.88 (3)	2.7472 (18)	173 (2)

Symmetry code: (i) $x - 1/2, -y + 3/2, z - 1/2$.

Table 2 Hydrogen-bond geometry (Å, º) for (izbt+24dhba)

D—H···*A*	*D*—H	H···*A*	*D*···*A*	*D*—H···*A*
N1—H1···O5^{i}	0.88	2.58	3.124 (3)	121
O2—H2···N2	0.84	1.8	2.633 (3)	175
O4—H4···O3	0.84	1.82	2.568 (3)	147
O5—H5···O1^{ii}	0.84	1.85	2.665 (3)	163

Symmetry codes: (i) $–x + 3/2, y + 1/2, −z + 1/2$; (ii) $x+ 3/2, −y + 1/2, z + 1/2$.

Table 3 Hydrogen-bond geometry (Å, º) for (izact + 1nta)

D—H···*A*	*D*—H	H···*A*	*D*···*A*	*D*—H···*A*
O2—H2*B*···N1	0.84	1.94	2.7758 (18)	179
N2—H2*A*···O1^{i}	0.88	2.07	2.9375 (17)	169

Symmetry codes: (i) $x + 1/2, −y + 3/2, z + 1/2$;

Table 4 Hydrogen-bond geometry (Å, º) for (izbt+2c4n)

D—H···*A*	*D*—H	H···*A*	*D*···*A*	*D*—H···*A*
N2-H2···O1^{i}	0.88(2)	2.05(2)	2.881(13)	158(2)
O2-H2*B*···N1	0.96(3)	1.65(3)	2.608(13)	173(2)

Symmetry codes: (i) x − 1/2, -y + 3/2, z − 1/2

Table 5 Hydrogen-bond geometry (Å, º) for (izbt+25dhba)

Table 6

D—H···*A*	*D*—H	H···*A*	*D*···*A*	*D*—H···*A*
O2—H2*B*···N1	0.84	1.76	2.596 (2)	173
O4—H4*A*···O3	0.84	1.84	2.577 (3)	146
O7—H7···N4	0.84	1.75	2.588 (3)	175
N2—H2*A*···O5^{i}	0.89 (3)	2.29 (3)	3.132 (3)	158 (2)
O5—H5*B*···O6	0.88 (3)	1.86 (3)	2.728 (2)	168 (3)
N5—H5*A*···O10^{ii}	0.90 (4)	2.34 (3)	3.178 (3)	156 (3)
O9—H9···O8	0.88 (5)	1.75 (5)	2.574 (3)	154 (5)
O10—H10···O1^{iii}	0.95 (4)	1.81 (4)	2.754 (2)	171 (3)

Symmetry codes: (i) −*x*+1, −*y*+1, −*z*+1; (ii) −*x*, −*y*, −*z*+1 (iii) *x*, *y*−1, *z*.

Table 7 S2 Powder Patterns

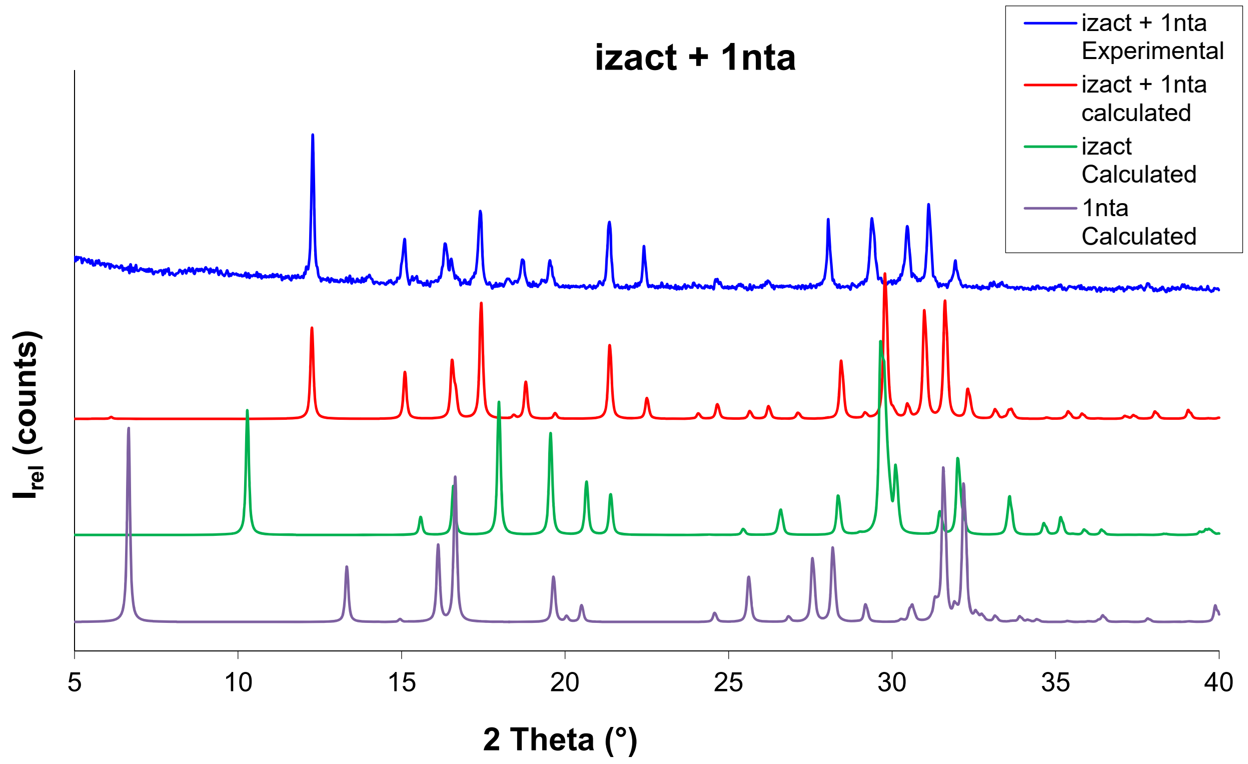

Figure S1 Powder pattern of the measured and calculated patterns of the cocrystal **izact + 1nta**.

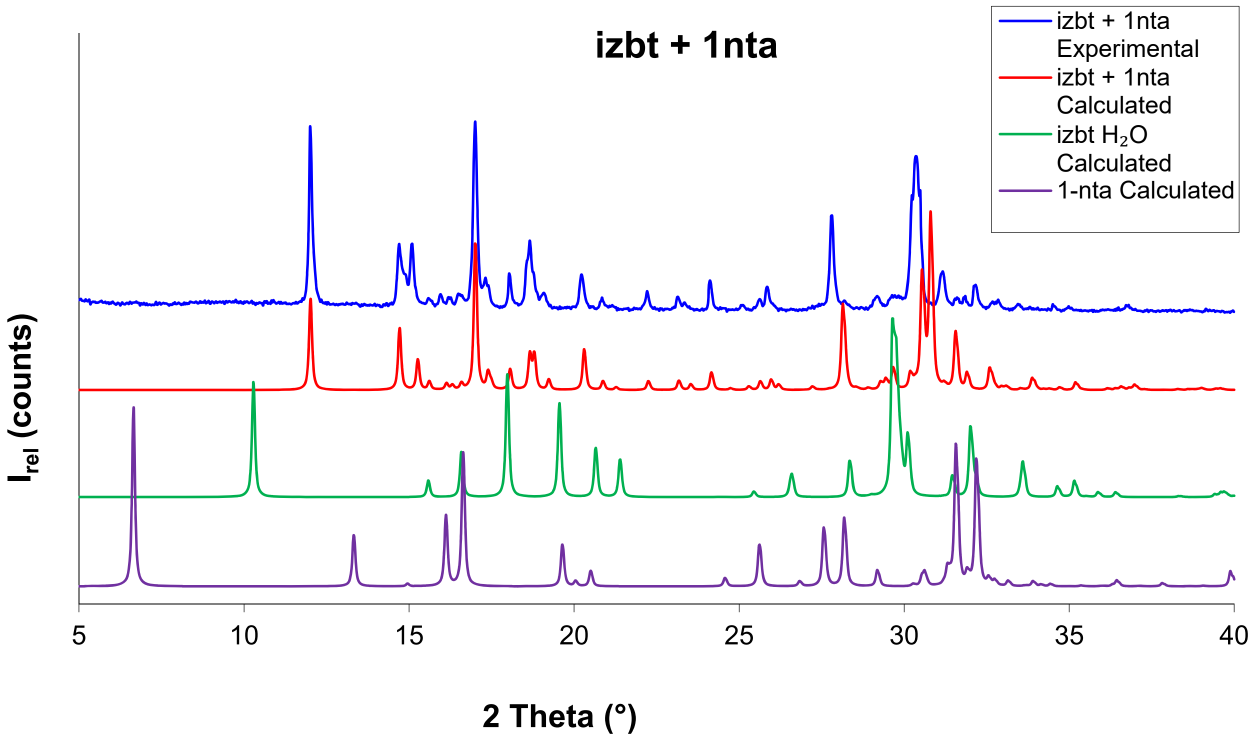

Figure S2 Powder pattern of the measured and₂ calculated patterns of the cocrystal **izbt + 1nta**.

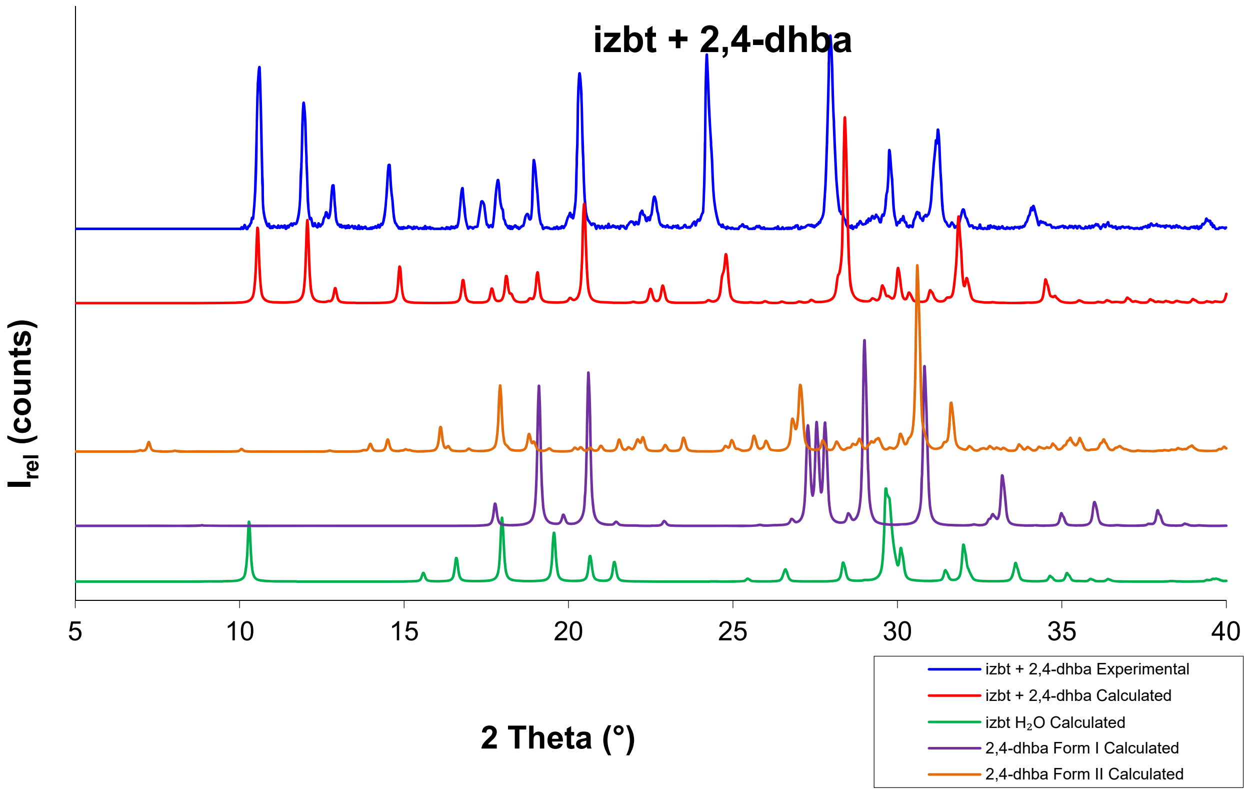

Figure S3 Powder pattern of the measured and calculated patterns of the cocrystal **izbt + 2,4-dhba**.

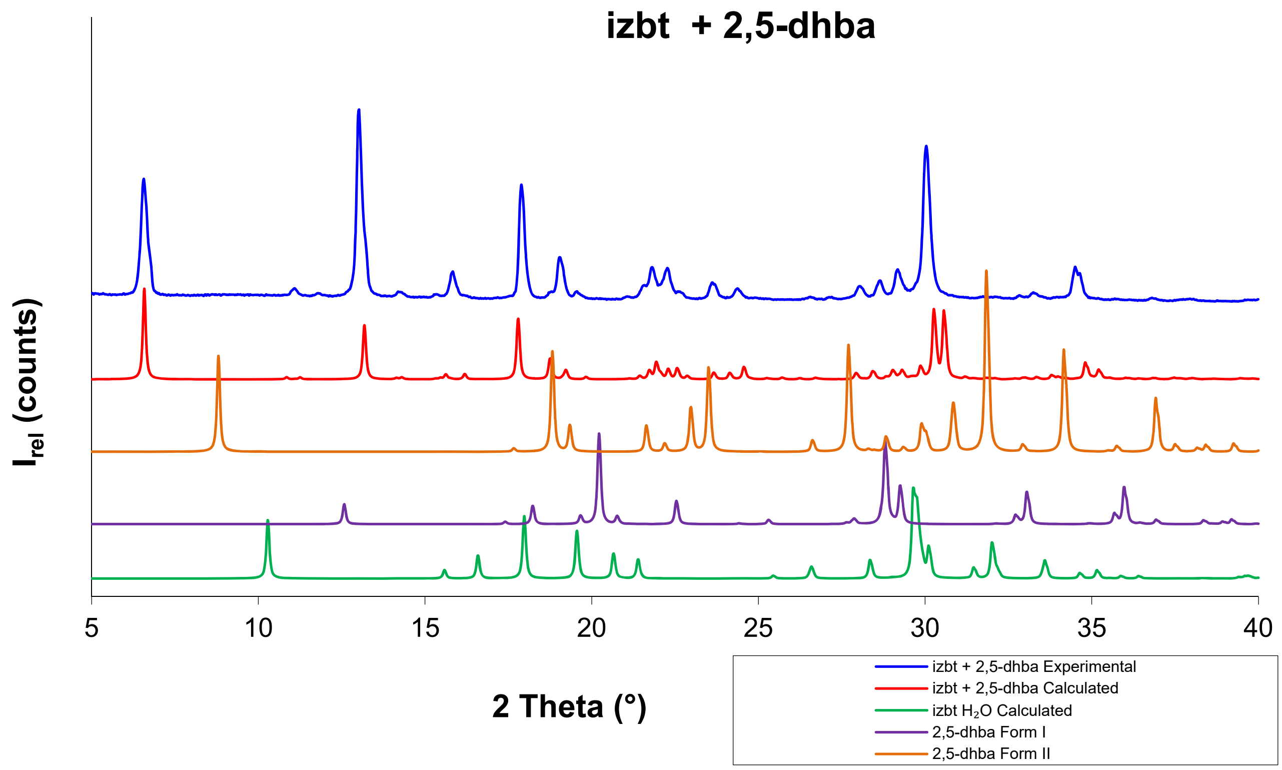

Figure S4 Powder pattern of the measured and calculated patterns of the cocrystal **izbt + 2,5-dhba**.

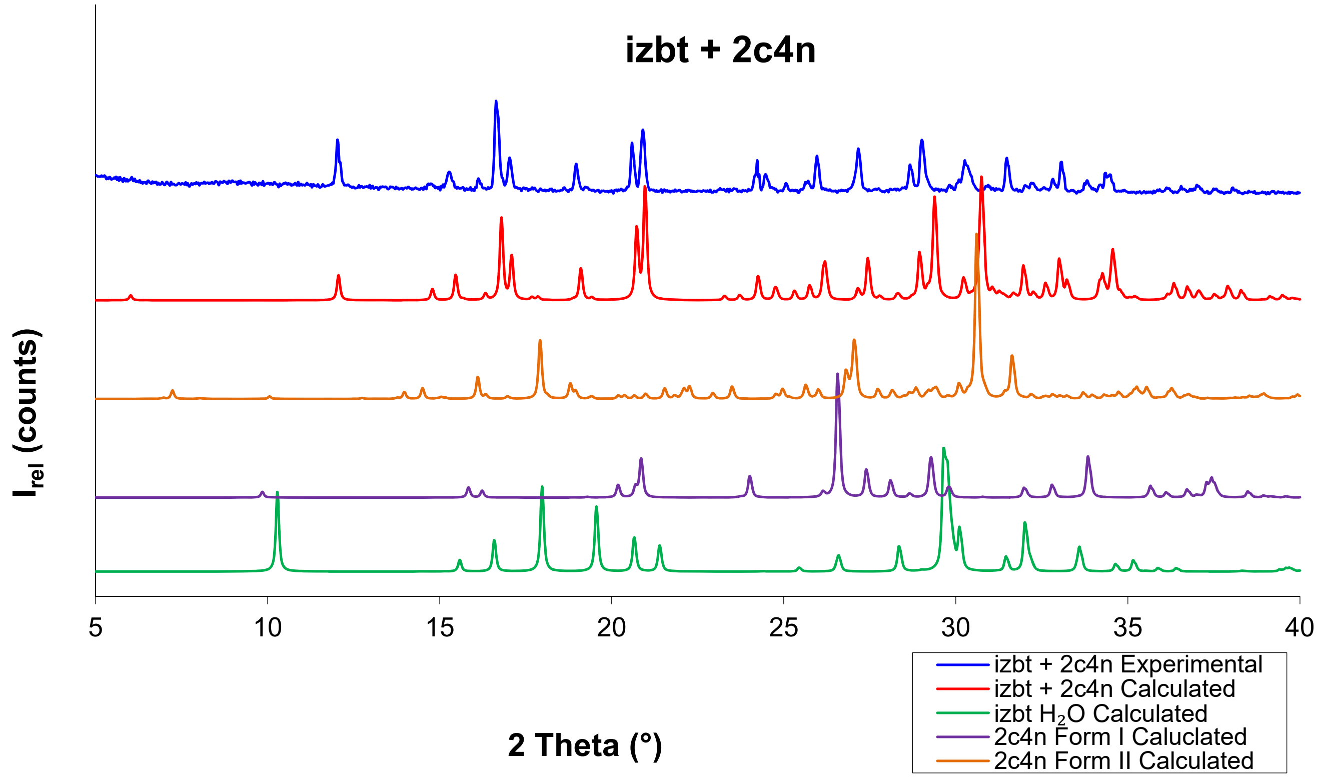

Figure S5 Powder pattern of the measured and calculated patterns of the cocrystal **izbt + 2c4n**.

S3 DSC curves

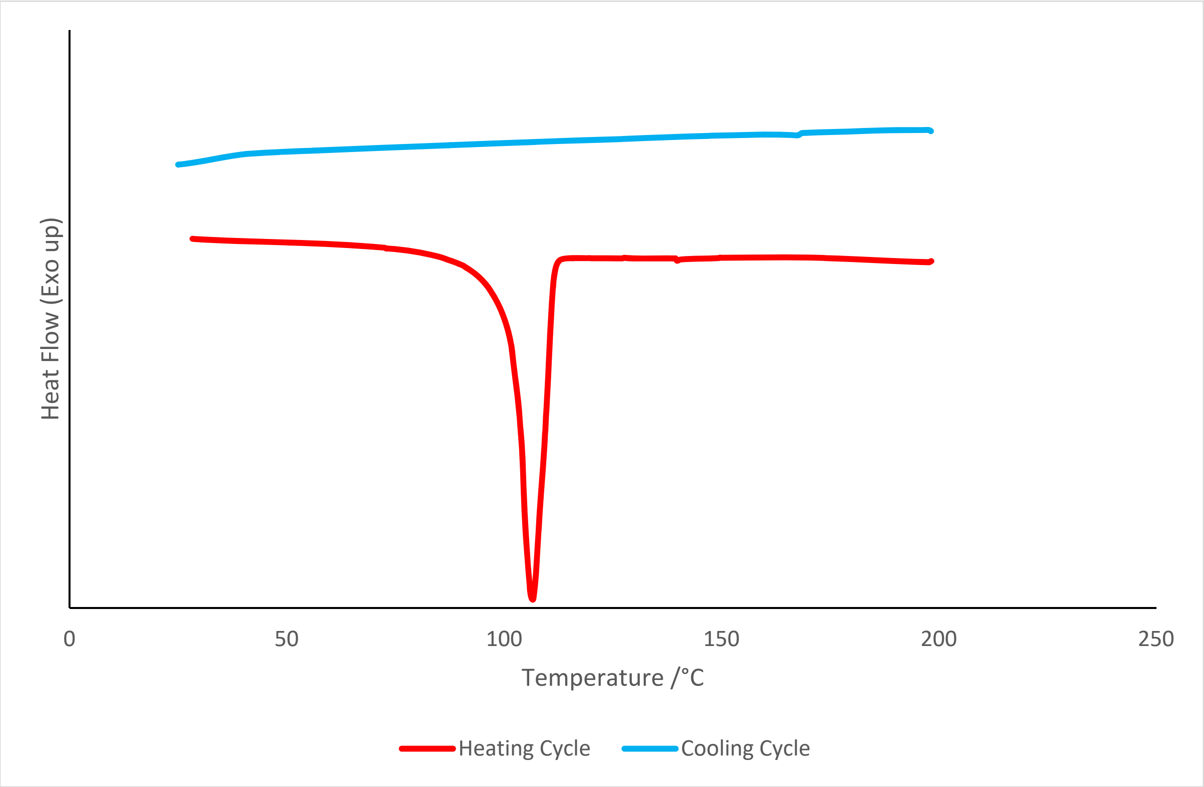

Figure S6 DSC curve of the cocrystal **izbt + 2c4n**.

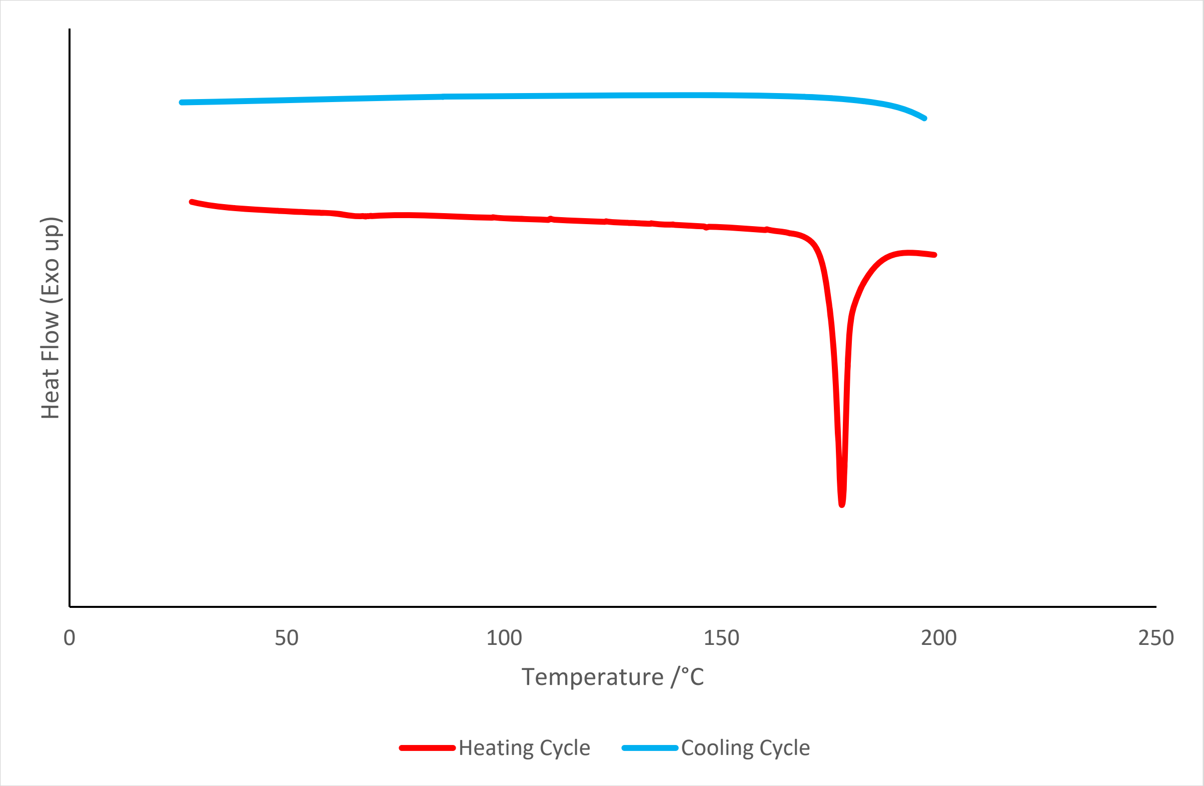

Figure S7 DSC curve of the cocrystal **izbt + 2,4-dhba**.

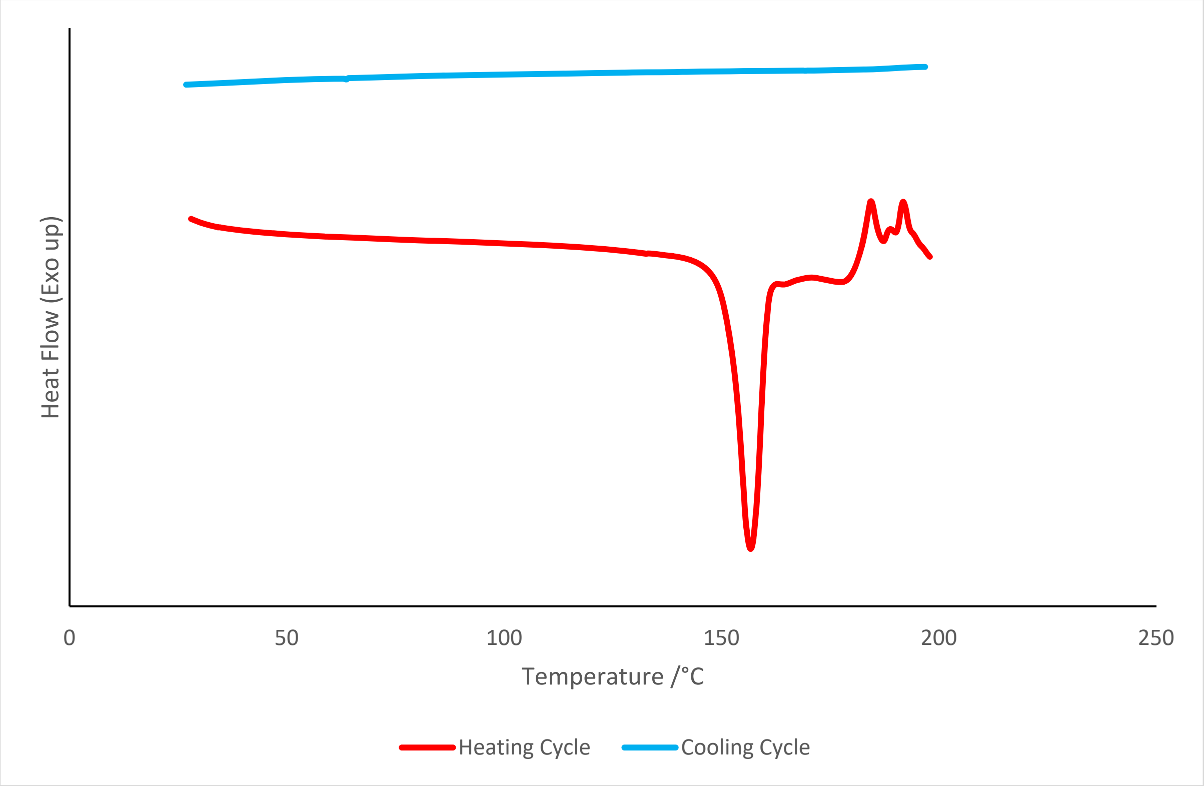

Figure S8 DSC curve of the cocrystal **izbt + 2,5-dhba**.

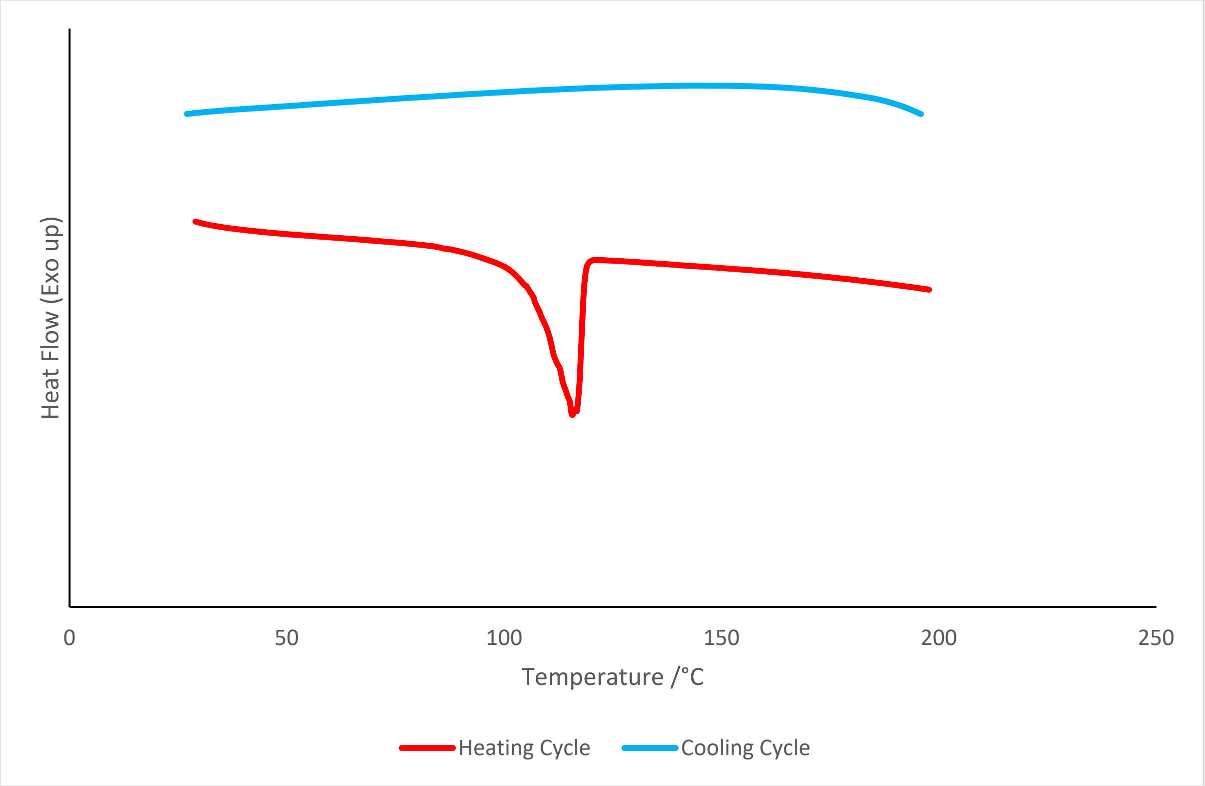

Figure S9 DSC curve of the cocrystal **izact + 1-nta**.
